# Exercise Combined with a Low-Calorie Diet Improves Body Composition, Attenuates Muscle Mass Loss, and Regulates Appetite in Adult Women with High Body Fat Percentage but Normal BMI

**DOI:** 10.3390/sports12040091

**Published:** 2024-03-25

**Authors:** Xinyue Wu, Chengnan Zhang, Zhuoying Liang, Yiheng Liang, Yuxuan Li, Junqiang Qiu

**Affiliations:** 1 Department of Exercise Biochemistry, Exercise Science School, Beijing Sport University, Beijing 100084, China; 2021210486@bsu.edu.cn (X.W.); 2023992990@bsu.edu.cn (C.Z.); liangzhuoying@bsu.edu.cn (Z.L.); yihengliang@bsu.edu.cn (Y.L.); 2021210478@bsu.edu.cn (Y.L.); 2 Beijing Sports Nutrition Engineering Research Center, Beijing 100084, China

**Keywords:** abnormal body fat percentage, caloric restriction combined with exercise, muscle mass loss, appetite

## Abstract

Background: The present study aimed to examine the effects of a 500 kcal reduction in daily energy intake alone and in combination with 90 min of moderate-to-vigorous aerobic exercise per week on body weight, body composition, and appetite sensations in young women with normal BMI and abnormal body fat percentage. Methods: sixty-six young women with normal BMI and abnormal body fat percentage (21.33 ± 1.20 kg/m^2^ and 34.32 ± 2.94%) were randomly assigned into three groups: (1) caloric restriction (CR; *n* = 22), (2) caloric restriction with exercise (CR–EX; *n* = 22), and (3) control (C; *n* = 22). Data on anthropometry, blood samples, and subjective appetite sensations pre- and post-intervention were collected. Results: After 4 weeks of intervention, CR and CR–EX groups both reduced body weight, fat percentage, and waist and hip circumferences compared to the C group (*p* < 0.05). Muscle mass of the CR group was significantly lower than that of the C group (−1.21 ± 0.86 kg vs. −0.27 ± 0.82 kg, *p* < 0.05), and no significant difference between CR–EX and C groups was observed. For appetite sensations, the subjects of the CR group showed significant increases in change of scores in desire to eat and prospective consumption than that of the C group (*p* < 0.05), while no significant difference between CR–EX and C groups was observed. Conclusion: A 500 kcal reduction in daily energy intake alone and in combination with 90 min of moderate-to-vigorous aerobic exercise per week could both reduce weight and improve body composition in young adult women with normal BMI and abnormal body fat percentage. More importantly, calorie restriction combined with exercise intervention was superior to calorie restriction alone in improving muscle mass loss and regulating appetite sensations.

## 1. Introduction

Body Mass Index (BMI) rather than body fat percentage is widely used as an indicator of obesity due to limited utilization of accurate body fat measurement techniques, such as dual-energy X-ray absorptiometry, which has led to many epidemiological and preventive strategy studies having limitations in obese populations with a normal BMI but elevated body fat percentage [1]. High body fat percentage has been shown to be closely associated with metabolic dysregulation [2]. The results of a general population cohort of middle-aged and older adults indicated that high body fat percentage was independently associated with increased all-cause mortality, suggesting the importance of research on the epidemiology and prevention strategies in populations with excess body fat percentage [3]. In particular, young Asian women with normal BMI but high body fat percentage may blindly adopt inappropriate weight-loss methods driven by the pursuit of slimmer bodies and weight-loss trends, leading them into a weight cycling trap. Survey data showed that increased body fat percentage was more prominent among young women who are dissatisfied with their weight, have planned or experienced weight loss, or have experienced fluctuating weight within one year [4,5]. Reliable supportive data are still needed to formulate rational strategies for reducing body fat percentage in people with high body fat percentage and normal BMI.

One of the important triggers of abnormal body fat percentage is the imbalance between energy intake and expenditure [6]. Caloric restriction is widely accepted as an effective method of preventing and treating obesity through lifestyle interventions [7]. The US Guidelines for the Management of Overweight and Obesity in Adults recommend controlling daily energy intake to between 1200 kcal and 1500 kcal for women and between 1500 kcal and 1800 kcal for men to prevent and treat obesity [8]. A study found that 70 obese individuals lost an average of 8.9 kg in weight and 4.4% in body fat percentage after 6 months of caloric restriction [9]. Another study reported that a reduction of 500 kcal in daily energy intake led to an average weight loss of 6.6 kg and a 14% reduction in body fat mass from baseline in obese adults after a 16-week intervention [10]. These findings suggested that caloric restriction could effectively promote a reduction in BMI and a decrease in body fat percentage in obese adults.

However, caloric restriction may cause problems such as muscle mass loss and increased appetite. Cava et al. reported that an 8–10% weight loss induced by caloric restriction was accompanied by a 2–10% reduction in muscle mass from baseline in obese individuals [11]. Moreover, caloric restriction was commonly paralleled by increased hunger and desire to eat, resulting in decreased long-term compliance with caloric restriction [12]. Exercise can alleviate muscle loss triggered by weight loss through a stimulatory effect on muscle anabolism [13]. Moreover, exercise promotes appetite regulation. The randomized controlled trial studies found that higher habitual physical activity could modulate the rise in appetite induced by energy deficits in obese adult and elderly populations [14,15]. Combined caloric restriction and exercise provides better improvements in blood glucose, insulin, triglyceride, as well as insulin resistance compared to single intervention in overweight and obese adults [16]. Given the potential synergistic effects of exercise and caloric restriction interventions, an increasing number of studies have focused on the effects of combined interventions on weight loss and health [17,18]. However, there are still few reports on weight loss efficacy and the health effects of combined caloric restriction and exercise interventions in young women with normal BMI but abnormal body fat percentage.

This study explored the combined effects of caloric restriction and caloric restriction combined with exercise on body weight, body composition, and appetite in young women with normal BMI and abnormal body fat percentage. This study aimed to provide scientific data for the development of rational strategies for a female population with weight management needs.

## 2. Materials and Methods

### 2.1. Subjects

A total of 70 young women (18–28 years old) were recruited from Beijing Sport University. All the volunteers were assessed according to the following inclusion criteria: (1) body fat percentage >33.3%; (2) body weight stable for 3 months prior to the study (weight gain or loss <4 kg); (3) no regular exercise (moderate-to-hard-effort exercise, ≥30 min/session, and three or more times per week); (4) no smoking, alcohol, or drug abuse; (5) no chronic diseases, no contraindications to exercise disease, no conditions that would interfere with interpretation of the results. Finally, 66 participants were selected for the present study.

### 2.2. Study Design and Randomization Procedures

The present study included a 4-week randomized controlled trial from May to June 2023 in Beijing, China. Subjects were randomly divided into one of the following three groups: group 1, only caloric restriction (CR; n = 22); group 2, caloric restriction combined with exercise (CR–EX; n = 22); group 3, Control (C; n = 22). Randomization was stratified by body weight to prevent any effect of an uneven distribution over the group allocation on outcomes.

**Figure 1:**
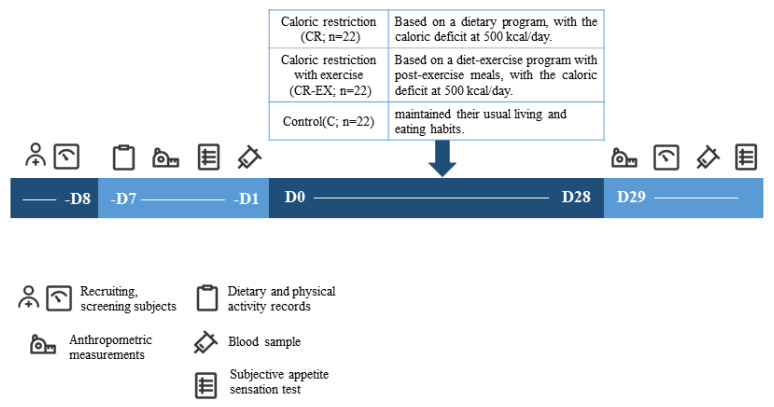
Experimental protocol.

During the 4-week intervention, subjects of CR and CR–EX groups received dietary intervention (caloric restriction) and diet–exercise joint intervention. Subjects of the C group maintained their usual living and eating habits throughout the entire study. All subjects were instructed to avoid consuming alcohol and caffeine and were asked to maintain their usual daily physical activity (Figure 1).

The study protocol was conducted in compliance with the Declaration of Helsinki and approved by the Internal Review Board of Beijing Sport University (No. 2023084H). Subjects were fully informed of the experimental procedures as well as potential risks and benefits of the study. Voluntary signed informed consent was received from all participating subjects before the intervention.

### 2.3. Interventions

#### 2.3.1. Dietary Intervention

Before the intervention, all the subjects were instructed to accurately record eating details during 3 days prior to the intervention. The average daily caloric intake of each subject was estimated by using a nutrient analysis application (Boohee, Shanghai, China). The utilization of the Boohee app for quantifying food intake has been considered as an effective dietary monitoring tool [19,20,21]. The 500 kcal caloric deficit for each subject in the CR and CR–EX groups was determined based on the estimated average daily caloric intake.

The dietary intervention in this study was mainly achieved through a customized diet (Pumpkin Health, Guangzhou, China). In order to satisfy the tastes of subjects, three choices of meals with a total energy ranging from 1000 kcal to 1300 kcal and macronutrient energy ratios of 20–25% for protein, 20–45% for fat, and 35–55% for carbohydrates were offered. An additional diet of 150 to 200 kcal, including skim milk, eggs, meal replacement biscuits, bananas, and sauces, was provided to enable subjects in CR and CR–EX groups to achieve the daily energy deficit of 500 kcal. In particular, subjects in the CR–EX group were additionally offered extra meals to compensate for the energy expenditure due to exercise. The diets were uniformly distributed by the researchers. The subjects in both groups reported their food intake details on a chat application every night (WeChat, Guangzhou, China).

#### 2.3.2. Exercise Intervention

The exercise intervention program was designed based on the WHO and the Physical Activity Guidelines for Chinese, which recommend that adults should perform at least 150 min of moderate-intensity or 75 min of vigorous-intensity exercise per week [22]. All the subjects in the CR–EX group were asked to complete the exercise intervention three times per week. Each exercise intervention consisted of a 5 min warm-up at a speed of 5 km/h and a 30 min aerobic running at a speed of 8 km/h on a running platform with an incline of 0°. Subjects were not allowed to engage in any other exercise intervention program except the one carried out in this study. The energy expenditure was measured by using three-axis accelerometers (Actigraph-GT3X, Pensacola, FL, USA).

### 2.4. Data Collection

#### 2.4.1. Anthropometric Measurements

Body weight was measured by a high-precision electronic scale (Balance, Xiamen, China), and BMI was calculated by dividing weight (kg) by the square of height (m). Waist circumference was measured at the narrowest portion of the waist, inferior to the xiphoid and superior to the iliac crests. Hip circumference was measured at the maximal posterior protuberance of the buttocks.

Body composition was assessed with dual-energy X-ray absorptiometry (GE Lunar IDXA, Madison, WI, USA) using the standard procedure for a full-body scan at the beginning and end of intervention. Using the enCORE version 15 software (GE Lunar, Madison, WI, USA), body fat percentage (BFP), body fat mass (BFM), and body muscle mass (BMM) were automatically calculated.

#### 2.4.2. Blood Analysis

Venous blood was collected in the morning after approximately 12 h overnight fasting and left to clot for 30 min at room temperature. The serum samples were obtained by centrifugation at 3000 rpm for 10 min at 4 °C and stored at −20 °C until analysis.

Serum ghrelin and PYY levels were quantified using enzyme-linked immunosorbent assay (ELISA) kits (Jianglaibio, Shanghai, China). Serum triglycerides, total cholesterol, high-density lipoprotein (HDL) cholesterol, low-density lipoprotein (LDL) cholesterol, and insulin levels were measured with a fully automatic immunoanalyzer (Beckman DXC 800, Fullerton, CA, USA). Fasting plasma glucose level (FPG) was measured by dry chemical method.

#### 2.4.3. Appetite Evaluation

Subjective appetite was measured using a visual analogue scale (VAS) in an early-morning fasting state at the beginning and end of the intervention. VAS consists of four dimensions, including desire to eat, hunger, satiety, and anticipation of eating. Subjects were asked to make a single vertical mark on a horizontal 10 -cm bar indicating an assessment of their current feelings from “not hungry at all” to “really hungry” [17]. VAS is a reliable tool for appetite research with good reproducibility, power, and validity [23].

### 2.5. Statistical Analysis

Power analysis was based on a previous study of the effects of the exercise with caloric restriction on body composition in overweight and obese subjects, which reported an effect size of 0.58 for changes in body weight index, with a power of 80%, and an α level (2-tailed) of 5% [24]. All analyses were performed using SPSS 26.0. The distribution pattern of the data was analyzed using the Kolmogorov–Smirnov test and presented as means ± SD. A two-factor, mixed-design analysis of variance (ANOVA) with a between-subject factor (group) and within-subject factor (time) was used to evaluate the differences between and within the groups for outcomes. *p*-values of <0.05 using 2-tailed tests were considered statistically significant. In case of a significant interaction, the simple main effects were tested and calculated to determine the difference between groups at each time point for each group. Otherwise, the main effects within the tests of within-subject effects were reported, and post hoc comparisons were performed via Tukey’s test.

## 3. Results

### 3.1. Study Overview and Subjects Characteristics

No adverse events, including fatigue, weakness, or loss of strength, were reported by subjects during the 4-week intervention. No significant differences in age, height, dietary calories, and physical activity energy expenditure among groups were observed at the beginning of the experiment (Table 1).

### 3.2. Energy Intake and Expenditure

After 4 weeks of intervention, the daily energy intake of the C group was significantly higher than those of CR and CR–EX groups (*p* < 0.05). The average daily energy intake during the intervention was reduced by 500.55 kcal in the CR group compared to the C group and by 104.73 kcal compared to the CR–EX group (Table 2). The average daily energy intake of the CR–EX group higher than that of the CR group resulted from additional diet given to compensate for the energy expenditure caused by exercise.

No significant difference in physical activities energy expenditure between the C and CR groups was observed (*p* > 0.05) (Table 2), indicating that subjects in C and CR groups maintained their physical activity habits during the 4-week period. Caloric difference between C and CR groups only resulted from the difference in dietary calories, which allowed comparison of the weight loss between C and CR groups at the same level of physical activity.

### 3.3. Weight and Body Composition

No significant differences in weight and body composition between groups were observed at the beginning and end of the intervention (Table 3). After the 4-week intervention, subjects in all groups showed significant decreases in weight, BMI and changes in weight and BMI (*p* < 0.05). The weight and BMI reductions in CR and CR–EX groups were significantly higher than that in the C group after the 4-week intervention (*p* < 0.05). 

After the 4-week intervention, body fat percentage, fat mass, and muscle mass of the CR–EX group (change in BFP: −1.49%, change in BFM: −1.55 kg, change in BMM: −0.71 kg) and the CR group (change in BFP: −1.45%, change in BFM: −1.45 kg, change in BMM: −1.21 kg) significantly decreased compared to baseline (*p* < 0.05). No significant difference between three groups in body fat percentage and fat mass was observed at the end of the intervention (*p* > 0.05). The changes of CR–EX and CR groups in body fat percentage and fat mass were significantly higher than that of the C group (*p* < 0.05). The body muscle mass of the CR group was significantly lower than that of the C group (*p* < 0.05), but no significant difference in body muscle mass of CR–EX and C groups was observed (*p* > 0.05).

Significant decreases in waist circumference and hip circumference of CR and CR–EX groups (*p* < 0.05), but not the C group (*p* > 0.05), were observed after 4 weeks of intervention. At the end of the intervention, the hip circumference of CR–EX and CR groups was significantly lower than that of the C group (*p* < 0.05). No significant differences in waist circumference were observed among groups (*p* > 0.05).

### 3.4. Blood Lipids and Fasting Plasma Glucose

Blood lipids and fasting plasma glucose of subjects at the beginning and end of the intervention are shown in Table 4. Hematological indices of all subjects before and after intervention were within the normal range, suggesting that the intervention did not negatively affect the health of subjects. No significant differences in blood lipid levels were observed among groups at the beginning and end of the intervention. The fasting plasma glucose levels of CR–EX and CR groups were significantly lower than that of the C group at the end of the intervention (*p* < 0.05), but no significant difference between CR–EX and CR groups was observed (*p* > 0.05).

### 3.5. Appetite Sensations

No significant differences in VAS scores between groups before intervention were observed. After intervention, scores of CR–EX and CR groups in hunger, desire to eat, and prospective consumption were significantly higher than that of the C group (*p* < 0.05). No significant difference in fullness scores among groups was observed (*p* > 0.05). For the changes in desire to eat and prospective consumption scores, the CR group was significantly higher than the C group (*p* < 0.05), but no significant difference was observed between CR–EX and C groups (*p* > 0.05). A significant increase in desire to eat score occurred in the CR group (*p* < 0.05) but not in CR–EX and C groups (*p* > 0.05). A significant increase in hunger scores occurred in the CR group (*p* < 0.05) but not in CR–EX and C groups (*p* > 0.05) (Table 5).

Appetite-regulating peptides levels of subjects at the beginning and end of intervention are shown in Table 6. No significant differences in the levels of insulin, ghrelin, and PYY were observed between groups before intervention. After intervention, a significant decrease in insulin levels (*p* < 0.05) occurred in the CR–EX group but not in CR and C groups (*p* > 0.05). PYY levels of the CR group were significantly decreased (*p* < 0.05). The insulin levels of the CR–EX group were significantly lower than those of the C group (36.40 pmol/L vs. 53.5 pmol/L; *p*< 0.05). No significant differences in ghrelin and PYY levels among groups after intervention were observed (*p* > 0.05).

## 4. Discussion

Obesity, a condition of abnormal or excessive fat accumulation within the body, has emerged as one of the severe public health issues worldwide. To our knowledge, there is no clear definition or diagnostic criteria to characterize abnormal body fat percentage. A general population cohort study found that body fat percentage was positively associated with all-cause mortality in adult women with a body fat percentage exceeding 33.3% [3]. The present study investigated the effects of a 4-week caloric restriction combined with exercise intervention on adult women with normal BMI and body fat percentage over 33.3%. The key findings of our study were as follows: (i) all caloric restriction groups experienced similar reductions in weight, BMI, body fat percentage, fat mass, waist circumference, and hip circumference over the intervention period; (ii) calorie restriction intervention caused a significant loss of muscle mass, whereas calorie restriction combined with exercise attenuated the loss of muscle mass; and (iii) calorie restriction caused elevated appetite as it became evident by a significant increase in changes of desire to eat and prospective consumption scores, while calorie restriction combined with exercise did not have a significant effect on appetite sensations.

Multiple practice guidelines suggest that a reduced-calorie nutritional plan and increased physical activity are recommended for all obese individuals [8,18,25]. Our results showed that reducing daily energy intake by 500 kcal through dietary control was effective in reducing body weight by more than 5%, even if additional meals were offered to compensate for the energy expenditure caused by exercise (Table 3), which is consistent with the findings of Tang et al. [26]. A 5% weight loss is widely recognized as the threshold at which clinically meaningful health improvements, including a decrease in systolic and diastolic blood pressure, fasting blood glucose, and glycated hemoglobin, as well as increase in high-density lipoprotein cholesterol, can be obtained [27,28]. However, most of the individuals were unable to achieve desirable weight loss via lifestyle intervention. Patients achieved an average weight loss of 3.6% after a 6-month period of nutritional and exercise counseling [29]. It is largely caused by the difficulty in complying with lifestyle change [30]. The present study suggested that desired weight loss could be achieved through a monitorable and rigorous lifestyle intervention with the help of standardized diets and wearable devices.

Although a weight-based BMI index is widely used in epidemiologic studies of obesity, one of its drawbacks is the inability to visually differentiate lean and fat mass. In contrast, body fat percentage allows for an intuitive evaluation of the body’s obesity status as well as the effectiveness of interventions [31,32,33]. The results showed that the body fat percentage and fat mass of caloric restriction groups significantly decreased by 1.45–1.55%, suggesting that caloric restriction that reduced body weight in both groups was partially attributed to the reduction in excessive fat accumulation (Table 3). Moreover, waist and hip circumferences of subjects in the calorie-restricted groups reduced by 2.48 to 3.14 cm (Table 3). A meta-analysis found that reducing caloric intake and increasing energy expenditure through exercise could both significantly reduce visceral fat mass [34]. Waist and hip circumferences can be used to reflect the degree of visceral fat accumulation [35]. Visceral fat content is an independent marker for the incidence of cardiovascular diseases [32]. Excessive accumulation of visceral fat content is associated with an increased all-cause mortality [36]. The reduction in waist circumference by 3 cm is considered to be clinically significant [37]. In addition, calorie restriction combined with an exercise intervention did not elicit an additional improvement in waist and hip circumferences in contrast to calorie restriction alone (Table 3), which was mainly attributed to the fact that the present study replenished the energy expenditure caused by the exercise by using an additional meal. These results indicated that caloric restriction was effective in improving body composition on adult women with normal BMI but abnormal body fat percentage.

Calorie restriction intervention is often accompanied by a reduction in fat-free mass [38]. Our results showed that calorie restriction alone caused a significant decrease in body muscle mass (Table 3). A negative energy balance may trigger enhanced whole-body proteolysis, amino acid oxidation, and nitrogen excretion and reduced muscle protein synthesis [39]. Exercise, including resistance and aerobic exercise, can promote muscle protein synthesis, restoration of intracellular lipid, and glycogen deposition in the liver and skeletal muscles [40]. Although resistance exercise is widely recognized to be more effective than aerobic exercise in increasing muscle mass, one study reported that no significant differences in total muscle mass amongst resistance training groups and controls when combined with dietary restriction resistance in obese adolescents were observed [41]. Our results suggested that caloric restriction combined with moderate-to-vigorous aerobic exercise could attenuate the muscle loss (Table 3), which was consistent with the findings of previous studies that synergy of diet and exercise appeared more effective in improving muscle loss than dietary approaches in isolation [42,43,44]. The present study demonstrated that caloric restriction combined with moderate-to-vigorous aerobic exercise can reduce weight while maintaining lean mass.

Reducing weight through caloric restriction alone may lead to compensatory eating behaviors [45]. Effects of habitual exercise on appetite control remain controversial, the discrepant results in appetite scores may be attributed to differences in the protocols used in different studies [46]. Our results indicated that calorie restriction combined with exercise interventions inhibit compensatory appetite rises due to daily energy intake deficits (Table 6), which is consistent with the findings of a study that found that a 2-week caloric restriction combined with exercise intervention reduced fasting hunger and maintained satiety in obese women [47]. Eating behaviors are influenced by several proposed mechanisms, one of which is appetite-related peptides. Both production of and response to many appetite-related peptides are impaired in obesity, resulting in the decreased capability to suppress hunger and appetite [48]. A greater suppression of appetite signals and greater stimulation of satiety signals are associated with higher-intensity exercise, suggesting that changes in appetite-regulating hormones following exercise appear to be intensity-dependent [49]. Our study did not find significant changes in ghrelin and PYY levels before and after the intervention, which may be due to the insufficient intensity of the exercise intervention used in the present study (Table 6). 

Obesity is one of the important causative factors for insulin resistance and type II diabetes mellitus. Dietary and exercise interventions have been widely incorporated as nonpharmacologic interventions in consensus guidelines for the prevention and treatment of type II diabetes mellitus [50,51]. Our results showed that both calorie restriction and calorie restriction combined with exercise interventions could significantly reduce insulin levels and fasting plasma glucose levels (Table 4 and Table 6), which is in line with the results of previous studies [50,51]. It is noteworthy that the fasting plasma glucose levels of the subjects were within normal levels before and after the interventions. Due to limitations, this pilot study had a relatively short intervention period and did not include participants with high body fat percentage and abnormal glycolipid or lipid levels. The effects of long-term calorie restriction combined with exercise on adult women with high body fat percentage and abnormal glycolipid levels deserve future research. Another limitation was that the present study enrolled only adult women; whether these findings are generalized to adult men warrant further study. Overall, our results suggested that a daily energy intake restriction of 500 kcal combined with 90 min of moderate-to-vigorous aerobic exercise per week could help adult women with normal BMI but abnormal body fat percentage achieve longer-term weight management and moderate the increase in appetite caused by the decrease in calorie intake to a certain degree.

## 5. Conclusions

The results of this study suggested that both caloric restriction and caloric restriction combined with exercise could reduce weight and BMI, improving body composition in adult women with normal BMI but abnormal body fat percentage. However, engaging in regular exercise based on caloric restriction with appropriate energy supplementation with additional meals to maintain a desirable energy deficit can improve muscle mass loss and regulate appetite sensations while reducing weight and fat mass. This suggests that combined interventions may be a favorable approach to long-term healthy weight loss for this population.

## Figures and Tables

**Table 1:** The baseline characteristics of subjects.

	CR–EX (n = 22)	CR (n = 22)	C (n = 22)	*p* Value
Age, years	20.77 ± 1.67	20.36 ± 1.22	21.05 ± 1.59	0.401
Height, m	163.36 ± 4.47	163.78 ± 4.20	165.91 ± 5.03	0.152
Energy intake, kcal/d	1835.57 ± 363.32	1819.18 ± 357.11	1829.96 ± 302.16	0.987
Physical activity energy expenditure, kcal/d	243.30 ± 50.18	218.22 ± 42.86	222.87 ± 44.40	0.320

CR, caloric restriction; CR–RS, calorie restriction with exercise; C, control. Differences among groups were evaluated using one-way ANOVA.

**Table 2:** Total energy intake and physical activity energy expenditure during intervention.

	CR–EX (n = 22)	CR (n = 22)	C (n = 22)	*p* Value
Energy intake, kcal/d	1452.48 ± 35.17 *$	1347.75 ± 30.28 *	1848.60 ± 357.6	<0.001
Physical activity energy expenditure, kcal/d	335.93 ± 40.94 *$	217.16 ± 38.95	219.22 ± 72.20	<0.001

CR, caloric restriction; CR–EX, calorie restriction with exercise; C, control. Differences among groups were evaluated using one-way ANOVA. An asterisk (*) indicates that the difference between groups (compare with C group) is significant (*p* < 0.05). A $ indicates that the difference between groups (compared with CR group) is significant (*p* < 0.05).

**Table 3:** Body weight and composition changes.

	CR–EX (n = 22)	CR (n = 22)	C (n = 22)	Group	Time	Group * Time
Weight, kg						
Baseline	57.59 ± 2.86	58.10 ± 2.95	57.10 ± 2.70	0.847	<0.001	<0.001
4 weeks	53.97 ± 2.87	54.17 ± 2.94	55.47 ± 3.14			
Change	−3.62 ± 1.09 *#	−3.94 ± 1.37 *#	−1.63 ± 1.09 *			
BMI, kg/m^2^						
Baseline	21.56 ± 1.04	21.61 ± 1.33	20.85 ± 1.07	0.279	<0.001	0.026
4 weeks	20.27 ± 0.99	20.19 ± 1.31	20.18 ± 1.20			
Change	−1.37 ± 0.43 *#	−1.47 ± 0.52 *#	−0.60 ± 0.39 *			
BFP, %						
Baseline	33.98 ± 2.66	35.56 ± 3.27	33.43 ± 2.64	0.196	<0.001	<0.001
4 weeks	32.49 ± 3.01	34.11 ± 3.74	33.42 ± 2.92			
Change	−1.49 ± 1.11 *#	−1.45 ± 1.10 *#	−0.01 ± 0.97			
BFM, kg						
Baseline	18.67 ± 1.75	19.72 ± 2.32	18.24 ± 1.71	0.270	<0.001	<0.001
4 weeks	17.12 ± 1.85	18.03 ± 2.22	18.13 ± 2.06			
Change	−1.55 ± 0.74 *#	−1.45 ± 1.10 *#	−0.11 ± 0.88			
BMM, kg						
Baseline	36.26 ± 2.31	35.64 ± 2.53	36.32 ± 2.39	0.689	0.033	0.584
4 weeks	35.56 ± 2.35	33.91 ± 2.39 $	36.05 ± 2.29			
Change	−0.71 ± 0.70 *	−1.21 ± 0.86 *#	−0.27 ± 0.82			
Waist circumference, cm						
Baseline	72.94 ± 4.11	73.63 ± 3.92	71.11 ± 3.08	0.804	<0.001	<0.001
4 weeks	70.36 ± 4.40	70.50 ± 3.90	71.46 ± 3.67			
Change	−2.58 ± 2.35 *#	−3.14 ± 2.18 *#	0.36 ± 1.94			
Hip circumference, cm						
Baseline	95.44 ± 2.48	95.51 ± 2.68	95.45 ± 2.62	0.130	<0.001	<0.001
4 weeks	92.85 ± 2.23 $	93.04 ± 2.62 $	95.75 ± 3.07			
Change	−2.60 ± 1.45 *#	−2.48 ± 1.94 *#	0.30 ± 1.90			

CR, caloric restriction; CR–EX, calorie restriction with exercise; C, control; BMI, body mass index; BFP, body fat percentage; BFM, body fat mass; BMM, body muscle mass. Differences between and within groups were evaluated using two-way ANOVA. An asterisk (*) indicates that the change in the index is significant (*p* < 0.05); a pound sign (#) indicates that the difference in the change between groups (compare with C group) is significant (*p* < 0.05); a $ indicates that the difference in the mean value between groups (compared with C group) is significant (*p* < 0.05).

**Table 4:** Intervention effects on blood lipids and fasting plasma glucose.

	CR–EX (n = 22)	CR (n = 22)	C (n = 22)	Group	Time	Group * Time
TG, mmol/L						
Baseline	3.92 ± 0.50	3.89 ± 0.68	4.10 ± 0.42	0.876	0.369	0.141
4 weeks	4.03 ± 0.85	3.96 ± 1.20	4.49 ± 0.73			
Change	−0.17 ± 0.65	−0.07 ± 0.21	0.09 ± 0.27			
TC, mmol/L						
Baseline	3.92 ± 0.50	3.89 ± 0.68	4.10 ± 0.42	0.155	0.032	0.276
4 weeks	4.03 ± 0.85	3.96 ± 1.20	4.49 ± 0.73			
Change	0.12 ± 0.76	0.07 ± 0.75	0.39 ± 0.61 *			
HDL-C, mmol/L						
Baseline	1.57 ± 0.27	1.59 ± 0.37	1.66 ± 0.27	0.306	0.007	0.150
4 weeks	1.62 ± 0.37	1.63 ± 0.50	1.83 ± 0.42			
Change	0.05 ± 0.30	0.03 ± 0.20	0.17 ± 0.21 *			
LDL-C, mmol/L						
Baseline	2.19 ± 0.53	2.18 ± 0.58	2.31 ± 0.47	0.438	0.015	0.643
4 weeks	2.33 ± 0.65	2.28 ± 0.83	2.56 ± 0.55			
Change	0.15 ± 0.58	0.10 ± 0.57	0.25 ± 0.46 *			
FPG, mmol/L						
Baseline	4.68 ± 0.40	4.75 ± 0.36	4.75 ± 0.31	0.004	0.895	<0.001
4 weeks	4.47 ± 0.43 $	4.60 ± 0.39 $	5.09 ± 0.51			
Change	−0.21 ± 0.38 #	−0.15 ± 0.42 #	0.34 ± 0.58			

CR, caloric restriction; CR–EX, calorie restriction with exercise; C, control; TG, triglyceride; TC, total cholesterol; HDL-C, high-density lipoprotein cholesterol; LDL-C, low-density lipoprotein cholesterol; FPG, fasting plasma glucose. Differences between and within groups were evaluated using two-way ANOVA. An asterisk (*) indicates that the change in the index is significant (*p* < 0.05); a pound sign (#) indicates that the difference of the change between groups (compare with C group) is significant (*p* < 0.05); a $ indicates that the difference in the mean value between groups (compared with C group) is significant (*p* < 0.05).

**Table 5:** Intervention effects on subjective appetite scores.

	CR–EX (n = 22)	CR (n = 22)	C (n = 22)	Group	Time	Group * Time
Hunger, cm						
Baseline	3.82 ± 2.74	3.55 ± 2.30	3.00 ± 2.14	0.006	0.034	0.069
4 weeks	4.73 ± 2.07 $	5.41 ± 2.38 $	2.68 ± 2.36			
Change	0.91 ± 2.94	1.86 ± 3.91 *	−0.32 ± 2.08			
Fullness, cm						
Baseline	2.09 ± 1.95	2.91 ± 2.45	2.95 ± 2.44	0.693	0.765	0.507
4 weeks	2.77 ± 2.37	2.64 ± 2.08	2.86 ± 2.88			
Change	0.68 ± 2.85	−0.27 ± 2.69	−0.09 ± 3.05			
Desire to eat, cm						
Baseline	3.95 ± 2.70	2.95 ± 1.79	3.23 ± 2.60	0.036	0.015	0.003
4 weeks	4.82 ± 2.54 $	5.18 ± 2.48 $	2.59 ± 1.89			
Change	0.86 ± 2.53	2.23 ± 3.09 *#	−0.64 ± 2.34			
Prospective consumption, cm						
Baseline	4.64 ± 1.81	4.00 ± 2.33	4.09 ± 1.85	0.057	0.001	0.018
4 weeks	5.64 ± 1.99 $	5.86 ± 1.36 $	4.05 ± 1.81			
Change	1.00 ± 2.12 *	1.86 ± 2.32 *#	−0.05 ± 2.06			

CR, caloric restriction; CR–EX, calorie restriction with exercise; C, control. Differences between and within groups were evaluated using two-way ANOVA. An asterisk (*) indicates that the change in the index is significant (*p* < 0.05); a pound sign (#) indicates that the difference in the change between groups (compare with C group) is significant (*p* < 0.05); a $ indicates that the difference in the mean value between groups (compared with C group) is significant (*p* < 0.05).

**Table 6:** Intervention effects on appetite-regulating peptides.

	CR–EX (n = 22)	CR (n = 22)	C (n = 22)	Group	Time	Group * Time
Insulin, pmol/L						
Baseline	53.45 ± 16.83	60.03 ± 20.39	50.08 ± 20.99	0.108	0.028	0.070
4 weeks	36.40 ± 12.63 $	49.05 ± 25.25	53.54 ± 28.08			
Change	−17.05 ± 22.00 *	−10.98 ± 34.90	3.45 ± 30.59			
Ghrelin, pg/mL						
Baseline	8.77 ± 4.00	9.17 ± 5.74	9.96 ± 2.81	0.943	0.447	0.343
4 weeks	9.69 ± 4.90	9.98 ± 5.86	9.33 ± 4.32			
Change	0.93 ± 4.01	0.82 ± 2.71	−0.64 ± 4.75			
PYY, pg/mL						
Baseline	8.34 ± 6.18	7.45 ± 4.25	9.11 ± 5.64	0.699	0.845	0.253
4 weeks	7.30 ± 2.78	8.68 ± 4.81	8.58 ± 4.51			
Change	−1.04 ± 4.95	1.23 ± 2.70	−0.53 ± 5.91			

CR, caloric restriction; CR–EX, calorie restriction with exercise; C, control. Differences between and within groups were evaluated using two-way ANOVA. An asterisk (*) indicates that the change in the index is significant (*p* < 0.05); a $ indicates that the difference in the mean value between groups (compared with C group) is significant (*p* < 0.05).

## Data Availability

Data are contained within the article.
